# Hydration as a governing state variable in the mechanical and electrical response of decellularized extracellular matrix materials

**DOI:** 10.1007/s10856-026-07075-6

**Published:** 2026-05-30

**Authors:** Xinyu Wang, Dawei Zhang, Maryam Hamed Al Jabri, Vincent Chan, Lianxi Zheng, Kin Liao, Peter R. Corridon

**Affiliations:** 1 Department of Biomedical Engineering and Biotechnology, College of Medicine and Health Sciences, Khalifa University of Science and Technology, Abu Dhabi, United Arab Emirates; 2 Healthcare Engineering Innovation Group, Khalifa University, Abu Dhabi, United Arab Emirates; 3 Department of Mechanical Engineering, Khalifa University of Science and Technology, Abu Dhabi, United Arab Emirates; 4 Center for Biotechnology, Khalifa University of Science and Technology, Abu Dhabi, United Arab Emirates; 5 Department of Aerospace Engineering, Khalifa University of Science and Technology, Abu Dhabi, United Arab Emirates; 6 Food Security and Technology Center (FSTC), Khalifa University, Abu Dhabi, United Arab Emirates

## Abstract

Decellularized extracellular matrix (dECM) materials derived from biological tissues continue to attract interest as sustainable biomaterials; however, their measured mechanical and electrical responses are highly sensitive to processing history and hydration state. While decellularization and subsequent lyophilization are widely employed to enable storage and handling, the role of hydration in governing experimentally measured dECM behavior remains insufficiently resolved. Here, hydration is examined as a governing state variable influencing the mechanical and electrical responses of dECM materials derived from ovine aortic tissue obtained from abattoir-sourced byproducts. Native, decellularized-lyophilized, and rehydrated samples were evaluated using uniaxial tensile testing, stress-relaxation analysis in circumferential and longitudinal orientations, and direct-current open-circuit potential (DC-OCP) measurements under defined testing conditions. Rehydration induced pronounced changes in early-strain stiffness, viscoelastic relaxation behavior, and electrical signal magnitude, indicating that water content and ionic environment dominate measured dECM responses. Comparable hydration-dependent trends were also observed in structurally distinct dECM tissues, supporting hydration state as a general materials variable rather than a tissue-specific effect. Importantly, the observed responses are interpreted as system-level interactions between dECM and its hydration environment, rather than recovery of native tissue properties. Collectively, these findings emphasize the need to explicitly control and report hydration conditions in dECM characterization and support a materials-centric framework for evaluating mechanical and electrical behavior in decellularized biomaterials.

**Graphical Abstract:**

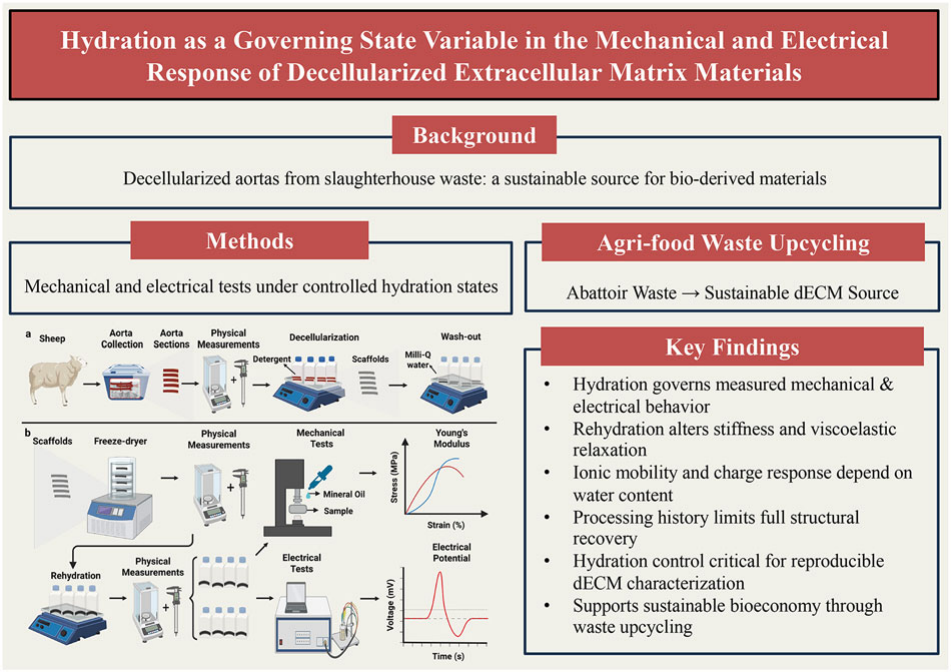

## Introduction

The aorta is critical for maintaining diastolic pressure, withstanding cyclic mechanical stress, and stabilizing blood flow [1]. Aging, diet, and congenital or acquired diseases such as atherosclerosis, aneurysms, and stenosis can irreversibly impair aortic function, often requiring surgical repair [2–5]. Decellularized aortic scaffolds derived from xenogeneic or allogeneic sources have therefore been widely explored as alternatives to synthetic grafts, owing to their complex extracellular matrix (ECM) composition and structural hierarchy [6].

However, generating decellularized ECM (dECM) materials at scale typically requires large quantities of animal tissues, raising ethical concerns and imposing significant economic burdens [7, 8]. In this context, slaughterhouse byproducts offer a sustainable and cost-effective alternative, aligning with circular bioeconomy principles and the growing demand for environmentally responsible biomaterials [9–12]. Global meat processing industries produce millions of tons of biological byproducts annually, representing a vast and underutilized resource for the derivation of ECM-based materials [10]. Leveraging this biomaterial stream not only reduces costs and purpose-bred animal tissues but also supports broader sustainability objectives, including alignment with the global Sustainable Development Goals (SDGs) related to health, responsible production, and climate action.

Anatomically, the aortic wall comprises three concentric layers, namely the tunica adventitia, tunica media, and tunica intima [13, 14]. Its structural integrity and hemodynamic compliance are governed by specialized ECM components, including collagen, which provides tensile strength, and elastin, which enables elastic recoil via the Windkessel effect [15–18]. The nonlinear stress-strain behavior of collagen/elastin networks underlies the stress-adaptive biomechanics of the aorta, reflecting the complex mechanical behavior of its specialized ECM [19, 20].

Beyond these fibrous proteins, glycosaminoglycans (GAGs) and proteoglycans (PGs) contribute to vascular mechanics through osmotic and electrostatic interactions, mechanisms that remain today as active areas of investigation [21]. Studies indicate that these components help maintain residual wall stress in unloaded conditions and that their individual and amalgamated contributions can be strongly influenced by hydration state and various solvents used during ex vivo testing [22–25].

In addition to mechanical behavior, the vascular ECM contributes to measurable electrical responses. Collagen exhibits piezoelectric behavior arising from asymmetric charge displacement during deformation [26, 27], with conductivity enhanced in hydrated environments. Elastin, while electrically insulating, can store charge during recoil and may participate in calcium-associated charge interactions [28–30]. GAGs differentially regulate ionic mobility and charge distribution, with hyaluronic acid enhancing ion transport via hydration, and sulfated GAGs modulating conductivity through fixed charge density [31–34]. Proteoglycans have been described as microcapacitive elements that contribute to both compressive mechanics and ionic behavior within the ECM [35, 36]. Collectively, these components give rise to hydration-sensitive electro-chemo-mechanical responses that can be probed experimentally under defined conditions [37].

Decellularization is widely employed to reduce tissue immunogenicity by removing cellular components, but it also alters the ECM at multiple structural and physicochemical levels [38, 39]. Chemical agents such as sodium dodecyl sulfate (SDS) and Triton X-100 have been shown to denature collagen, disrupt crosslinking, and impair lamellar organization, thereby altering tensile behavior [40]. Elastin networks can similarly be affected by aggressive chemical treatments, diminishing elastic recoil [41]. Highly soluble GAGs are often depleted during decellularization, altering hydration-dependent mechanics and ionic interactions [42, 43]. Comparatively, proteoglycans, particularly sulfated species, are vulnerable to chemical and enzymatic degradation [44, 45]. Ionic environments introduced during processing can further influence ECM structure and electrical response, contributing to variability in measured outcomes [46].

Rather than viewing these changes solely as a loss of native tissue function, there is increasing recognition that the dECM should be treated as a hydration-sensitive material system, whose experimentally measured properties depend strongly on processing history and environmental state. Numerous strategies have been explored to modify or augment dECM behavior, including polymeric coatings, biochemical supplementation, crosslinking, and recellularization approaches [47, 48]. However, many of these strategies introduce additional complexity, cost, and variability.

**Fig. 1:**
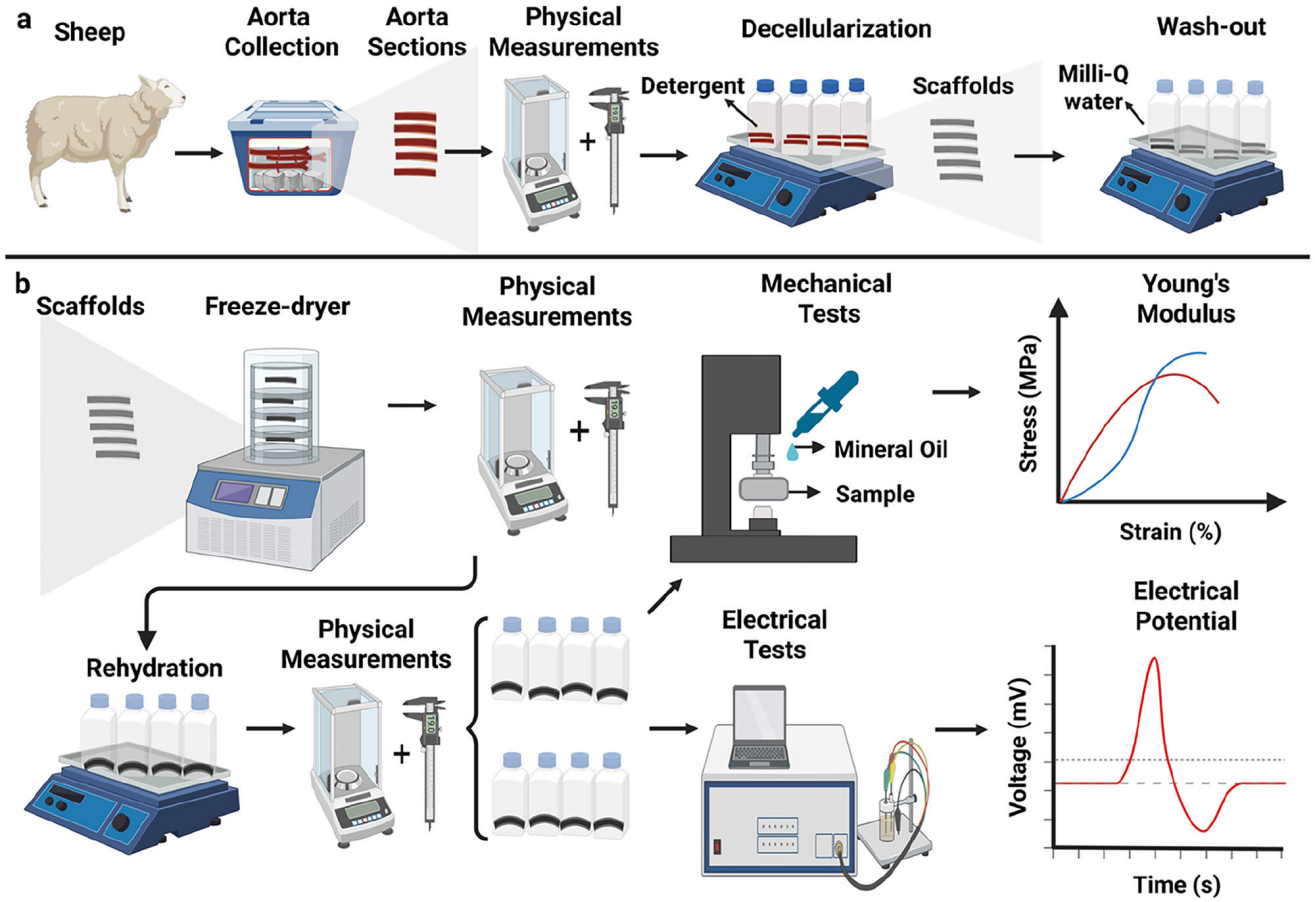
Experimental workflow illustrating the use of abattoir-sourced byproducts. This approach was used to examine hydration-dependent mechanical and electrical responses of aortic dECM materials. **a** Decellularization and wash-out process applied to ovine aortas obtained from slaughterhouse byproducts. **b** Lyophilization and subsequent rehydration of aortic dECM, followed by mechanical testing and DC-OCP measurements under defined testing conditions

Accordingly, this study treats hydration as a governing state variable that shapes the mechanical and electrical behavior of ovine aortic dECM materials sourced from slaughterhouse byproducts. Decellularization was performed using a detergent-based protocol previously reported by our group, followed by lyophilization and controlled rehydration. Mechanical behavior was assessed through uniaxial tensile and stress-relaxation testing in circumferential and longitudinal orientations. Likewise, electrical measurements were conducted using direct-current open-circuit potential (DC-OCP) measurements under defined ionic conditions. By comparing native, lyophilized, and rehydrated states, this work focuses on the hydration-dependent modulation of measured responses rather than on the restoration of native tissue properties. Additional measurements in structurally distinct dECM tissues were used to assess the generality of hydration-mediated trends. Collectively, our materials-centric framework provides insight into how hydration state governs experimentally measured mechanical and electrical responses in dECM systems, derived from sustainable agri-food byproduct streams. This framework is illustrated in Fig. 1.

## Materials and methods

### Aorta collection

**Fig. 2:**
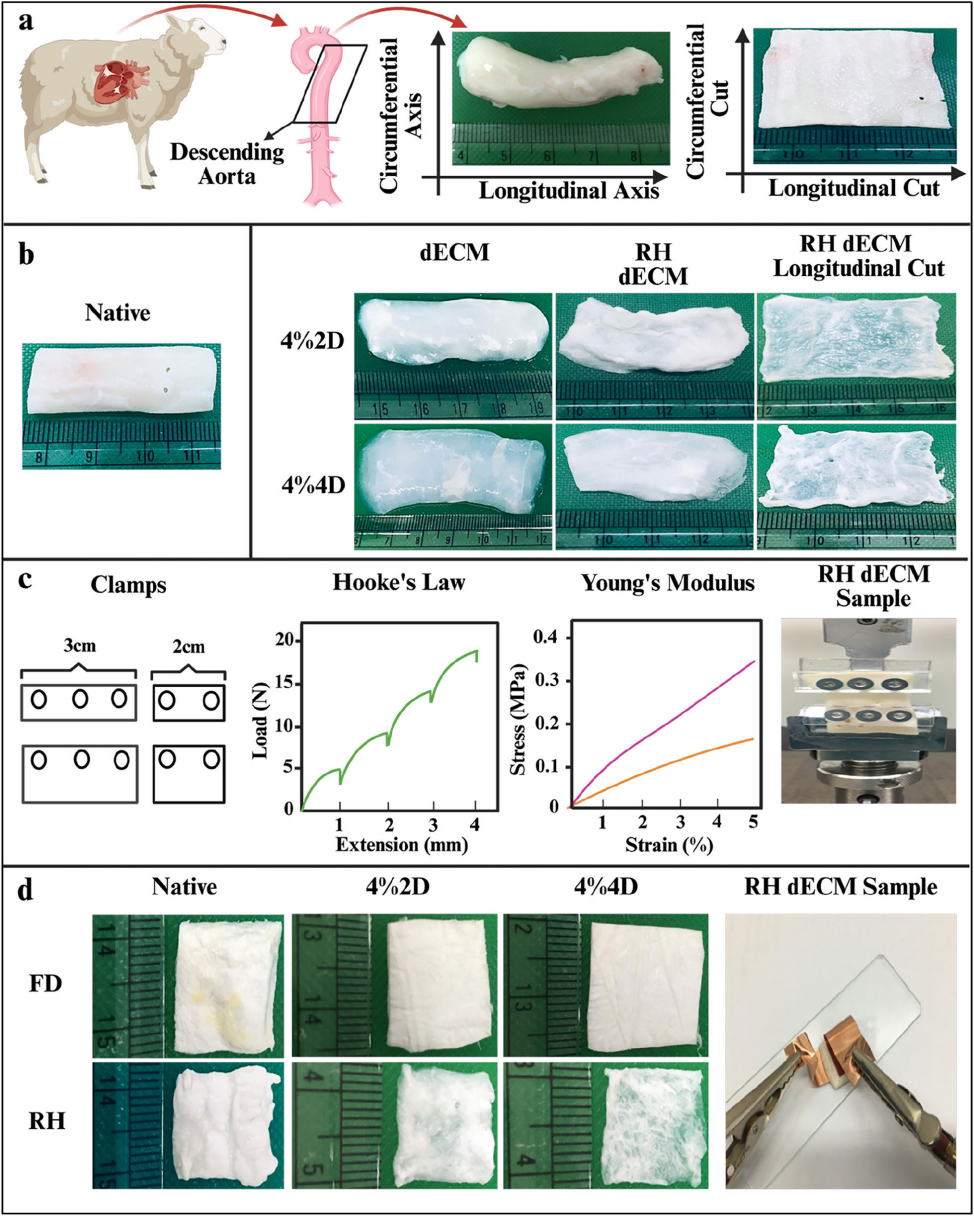
Sample preparation, processing states, and experimental configuration. **a** Segmentation of ovine aortas obtained from agri-food-derived byproducts and preparation of samples for analyses. **b** Representative macroscopic images of aortic samples in native, decellularized, freeze-dried, and rehydrated states. **c** Experimental configuration for uniaxial tensile and stress-relaxation testing in circumferential and longitudinal orientations, including custom 3D-printed clamps. **d** Experimental configuration for electrical measurements, showing electrode placement on the adventitial and intimal surfaces under defined hydration conditions. Abbreviations: NT native tissue, dECM decellularized extracellular matrix, FD freeze-dried, RH rehydrated, A adventitia, I intima

Fresh discarded ovine aortas were obtained from the Abu Dhabi Automated Slaughterhouse and immediately stored in saline containing antibiotics on ice within a cooler box. The tissue segments were transported to Khalifa University of Science and Technology within approximately 30–60 min of extraction. Upon arrival, the descending thoracic aorta was isolated from the whole aorta and segmented into pieces approximately 4 cm in length (Fig. 2a). Excess adipose tissue surrounding the aortic segments was carefully removed to ensure consistency for subsequent processing.

### Sample grouping and decellularization protocol

Aortic samples were categorized into three groups: native, 4% 2D, and 4% 4D. Native samples were stored in 0.9% saline with antibiotics at 4 °C until testing. The 4% 2D and 4% 4D groups were decellularized using a 4% detergent solution (Ecover, Malle, Belgium) for 2 days and 4 days, respectively, under continuous agitation at 300 rpm using an Ohaus™ Analog Heavy Duty Shaker (ThermoFisher Scientific, Waltham, MA, USA). The detergent solution consisted of a commercially available non-ionic/anionic formulation (Ecover), containing surfactants functionally analogous to Triton X-100 and SDS, enabling disruption of cellular membranes and removal of intracellular components.

The detergent-based protocol employed here has been previously reported by our group for ECM processing and was not optimized in this study. Each experimental group comprised 18 independent aortic segments obtained from different animals (biological replicates, *n* = 18). Six samples were allocated for mechanical testing in both circumferential and longitudinal directions, while the remaining samples were used for electrical characterization. At the end of the decellularization period, samples underwent a wash-out process using Milli-Q deionized water over three days, with the water replaced three times daily. Following wash-out, samples were stored in Milli-Q water at 4 °C prior to freeze-drying and subsequent testing. All reported sample numbers (n) represent independent biological replicates obtained from distinct animals unless otherwise specified.

### Freeze-drying and rehydration protocols

Eighteen samples from each decellularized group were freeze-dried for 24 h using a Labfirst Scientific laboratory freeze-dryer (FDL series, Shanghai, China). Fifteen samples from each group were subsequently rehydrated by immersion in Milli-Q deionized water for 24 h under continuous agitation at 300 rpm. Of the rehydrated samples, 12 were designated for mechanical testing and three for electrical measurements. In addition, three non-rehydrated (freeze-dried) samples from each group were used for electrical testing. For native tissue, 12 non-freeze-dried samples were used for mechanical testing, while three native and three freeze-dried samples were allocated for electrical measurements. All freeze-dried samples were stored in sterile petri dishes sealed with parafilm in a dry environment, while rehydrated samples were maintained in Milli-Q water at 4 °C until testing. Representative images are shown in Fig. 2b. Histological, mechanical, and electrical measurements were performed on independent sample sets.

### Physical change measurements

Weight, thickness, and diameter of independent aortic segments (biological replicates, *n* = 24 per group) were measured to assess physical changes associated with decellularization, freeze-drying, and rehydration. An analytical balance (US Solid, CA, USA) and a digital caliper (Mitutoyo 500-197-30, Boston, MA, USA) were used. Hydrated samples were gently blotted to remove excess surface liquid prior to measurement, while freeze-dried samples were measured directly. These samples represent an independent cohort from those used in mechanical and electrical testing, resulting in a larger sample size for physical measurements (*n* = 24 per group) compared to other analyses.

### Histological analysis

**Fig. 3:**
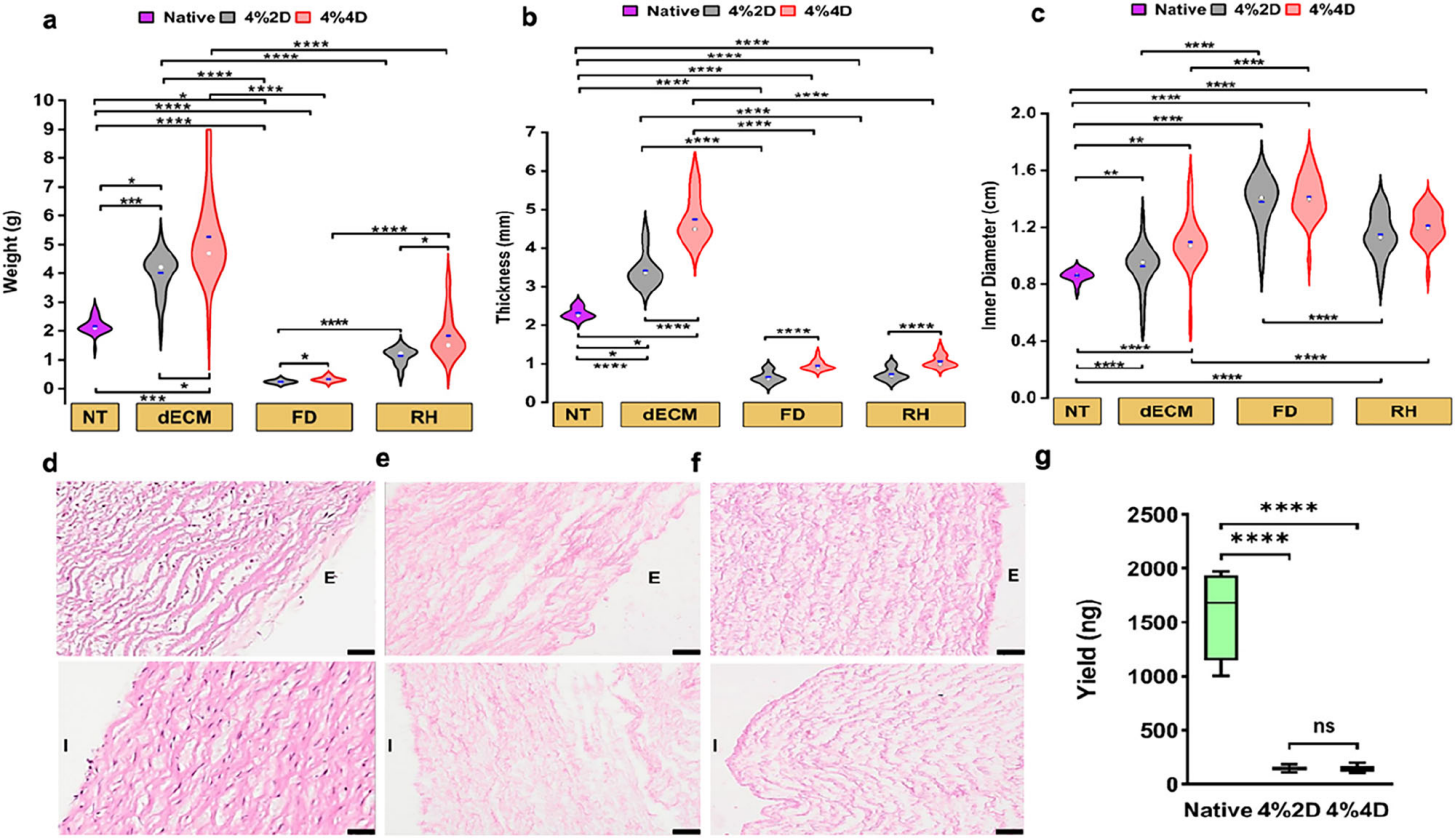
Physical measurements and decellularization validation. Physical and compositional characterization of aortic dECM materials across processing states. **a** Weight measurements of aortic samples in native, decellularized, freeze-dried, and rehydrated states. **b** Aortic sample thickness across the same processing conditions. **c** Inner diameter measurements of aortic samples across processing states. **d** H&E staining of native aortic tissue. **e** H&E staining of aortic tissue decellularized using the 4%2D protocol. **f** H&E staining of aortic tissue decellularized using the 4%4D protocol. **g** Quantification of residual DNA content following decellularization. Abbreviations: NT native tissue, dECM decellularized extracellular matrix, FD freeze-dried, RH rehydrated, A adventitia, I intima. Sample sizes: *n* = 24 per group for physical measurements; *n* = 3 per group for DNA quantification

Bright-field micrographs were acquired using an Olympus CX43 microscope at 20× magnification. The tunica adventitia (TA) and tunica intima (TI) were examined (Fig. 3d–f). Samples were fixed in 10% neutral-buffered formalin for 1 h at 4 °C, rinsed and dehydrated through graded ethanol. The tissues were subsequently cleared in xylene, embedded in paraffin, and sectioned at 4 µm. Sections were stained with hematoxylin and eosin (H&E) for qualitative structural assessment. Histological analysis was performed on three independent samples per group (*n* = 3).

### DNA quantification

DNA quantification was performed to confirm removal of cellular material following decellularization, but was not used as a primary outcome measure of material performance. Genomic DNA was extracted using a QIAamp DNA Mini Kit (Qiagen, Germantown, MD, USA) according to the manufacturer’s instructions and established protocols [49–52]. DNA concentration and purity were assessed using a Nanodrop Microvolume Spectrophotometer (ThermoFisher Scientific, Waltham, MA, USA), and integrity was evaluated via 1% agarose gel electrophoresis.

### Mechanical testing

Custom clamps were designed and fabricated using a 3D printer (Fig. 2c). Uniaxial tensile testing was performed using an Instron 5900 Series Universal Testing System (Norwood, MA, USA) at a strain rate of 0.1 mm s⁻¹, with tests terminated at 60 N. Six independent samples per group (biological replicates, *n* = 6) were tested in both circumferential and longitudinal orientations. To minimize evaporation and preserve hydration state during testing, samples were continuously coated with a thin layer of mineral oil, as previously validated for hydrated aortic tissues [53]. Stress-relaxation tests were performed by sequential loading (0, 5, 15, and 20 N), with each extension held for approximately 5 min while load decay was recorded.

### Electrical measurements

Electrical measurements are reported as hydration-dependent DC-OCP responses under defined testing conditions. Square specimens (1 cm²) were excised from each sample (Fig. 2d). For each decellularized group, three independent biological samples per condition (*n* = 3) were tested, while native samples were evaluated in fresh and freeze-dried states. Copper electrodes were placed on the TA and TI surfaces, and DC-OCP measurements were conducted using an Autolab PGSTAT302N Potentiostat/Galvanostat Unit (Metrohm, Switzerland). To assess hydration effects during testing, sample mass loss due to evaporation was monitored over time (*n* = 3 per group).

### Ethical declarations

Ovine aortic tissues were obtained as discarded byproducts from the Abu Dhabi Automated Slaughterhouse following routine food-processing procedures. Tissue collection did not involve any additional animal handling or sacrifice for research purposes. All procedures were approved by the Animal Research Oversight Committee (AROC) at Khalifa University of Science and Technology.

## Results

### Physical change measurements

Changes in weight, thickness, and inner diameter of aortic samples in native, decellularized, freeze-dried, and rehydrated states across the three experimental groups (native, 4% 2D, and 4% 4D) are shown in Fig. 3a–c. Violin plots with Kruskal-Wallis analysis (*P* **<** 0.05) revealed significant differences in sample weight across processing states within both decellularized groups. Compared to native tissue, rehydrated dECM samples, particularly in the 4%4D group, did not exhibit statistically significant differences in mass (*P* **>** 0.05).

Thickness measurements showed significant differences between the 4%2D and 4%4D groups under identical processing conditions (*P* **<** 0.05). Within each group, freeze-dried and rehydrated samples did not differ significantly in thickness (*P* **>** 0.05).

Inner-diameter measurements showed significant variation across most state-wise comparisons (*P* **<** 0.05). Representative images (Fig. 2b) show that rehydrated dECM samples exhibited increased wall thickness and reduced luminal diameter relative to native tissues.

### Decellularization assessment

Representative H&E staining (Fig. 3d–f) illustrates structural features of native and decellularized aortic tissues. Native samples displayed visible cell nuclei within the tunica adventitia and endothelial cells lining the tunica intima. In contrast, decellularized samples from both 4% 2D and 4% 4D groups showed the absence of visible nuclei.

DNA quantification (Fig. 3g) demonstrated greater than 90% reduction in DNA content in both decellularized groups relative to native tissue (*P* < 0.05). No statistically significant difference was observed between the 4%2D and 4%4D protocols (*P* > 0.05).

### Sustainability of abattoir-derived tissue sourcing

Approximately 4 kg of ovine aortic tissue was collected, of which 360–420 g was used in the present experiments. Additional portions were simultaneously utilized across multiple research projects, resulting in near-complete utilization of the collected tissue.

Based on reported emissions of 3–4 kg CO₂ per kilogram of organic byproduct, repurposing the collected tissue avoided an estimated 12–16 kg of CO₂ emissions.

### Mechanical response

**Fig. 4:**
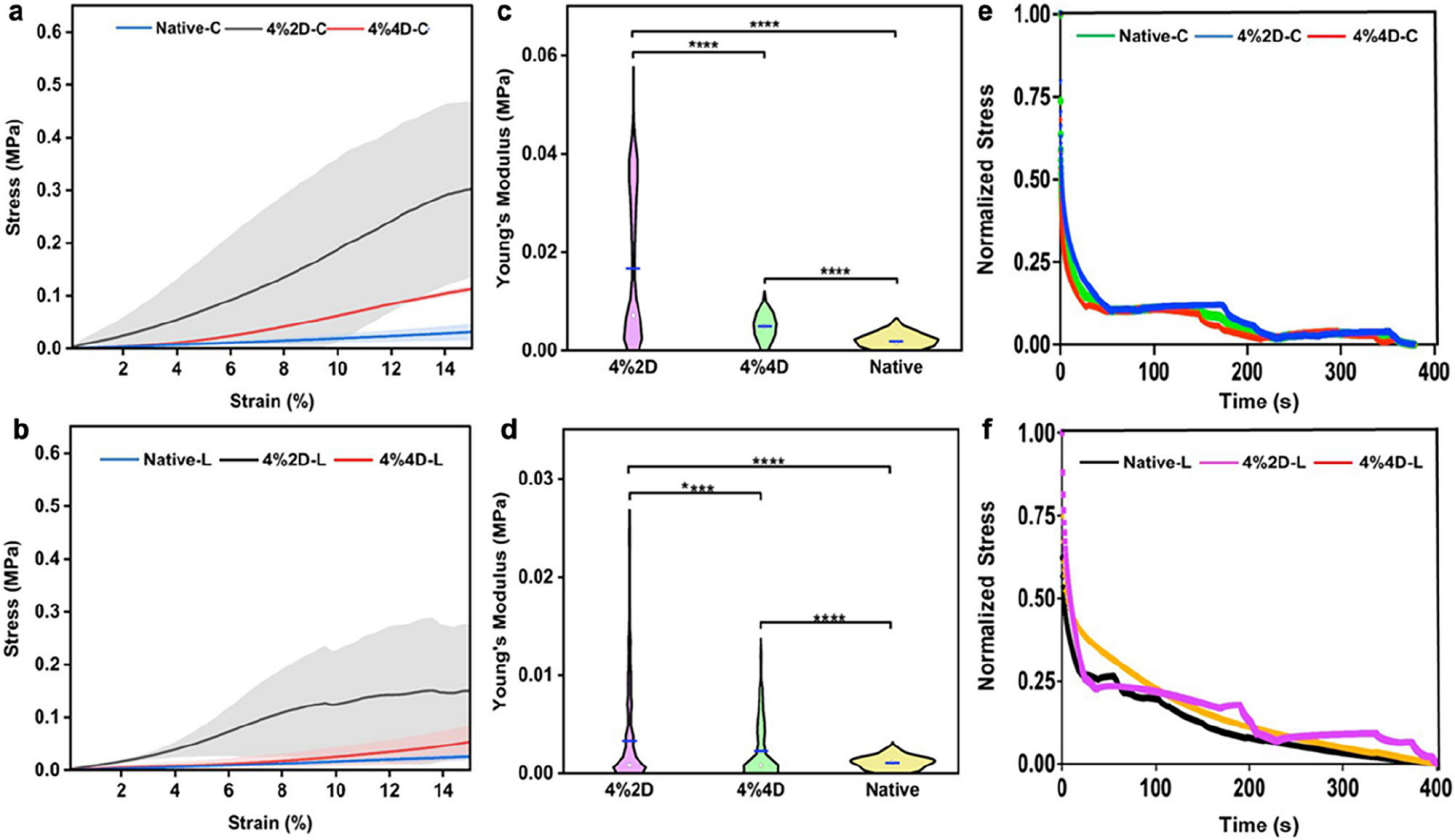
Tensile and stress-relaxation characterization of aortic dECM. Mechanical response of rehydrated aortic dECM materials under uniaxial loading and stress-relaxation. **a** Stress-strain response under circumferential loading. **b** Stress-strain response under longitudinal loading. **c** Young’s modulus calculated from circumferential loading. **d** Young’s modulus calculated from longitudinal loading. **e** Log-normalized stress-time response during stress-relaxation under circumferential loading. **f** Log-normalized stress-time response during stress-relaxation under longitudinal loading. Abbreviations: C circumferential, L longitudinal. Sample sizes: biological replicates, *n* = 6 per group per orientation for tensile testing; *n* = 3 per group per orientation for stress-relaxation testing

Stress-strain responses of rehydrated dECM under 15% strain in circumferential and longitudinal orientations are shown in Fig. 4a, c. Rehydrated 4% 2D and 4% 4D samples exhibited higher stress at equivalent strain levels compared to native tissue.

Young’s moduli differed by orientation (Fig. 4b, d). At low strain (0-5%), circumferential stiffness exceeded longitudinal stiffness across all groups. At higher strain (5-10%), circumferential stiffness remained greater. The Kruskal-Wallis test presented significant differences in modulus among groups (*P* < 0.05).

Measured moduli of rehydrated dECM remained below reported physiological arterial stiffness ranges.

Stress-relaxation behavior (Fig. 4e, f) showed differences in normalized stress decay profiles among groups (*P* < 0.05). Rehydrated dECM samples exhibited reduced stress decay and higher plateau levels relative to freeze-dried samples.

### Electrical measurements

**Fig. 5:**
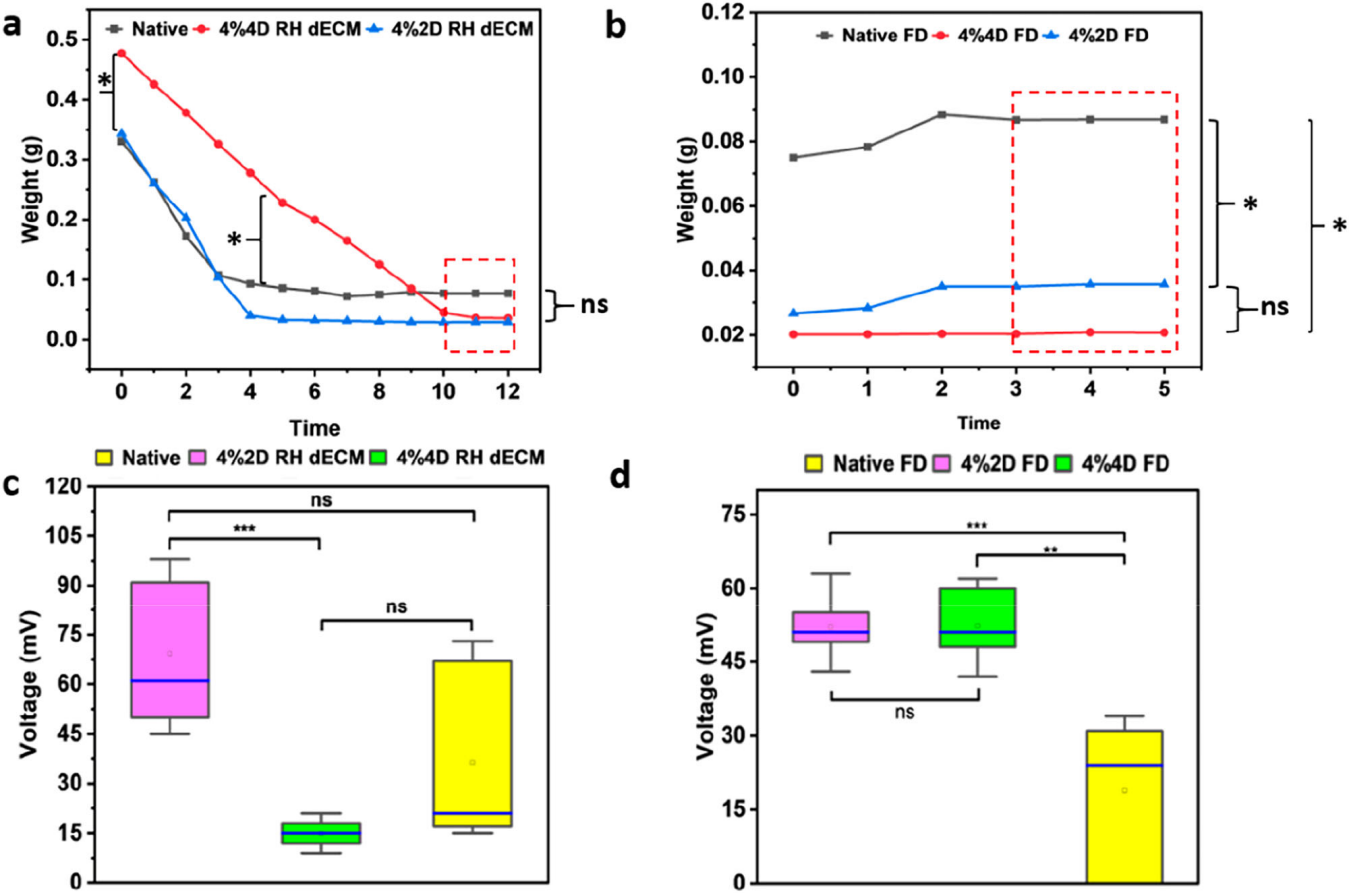
Hydration-dependent electrical measurements of aortic dECM. Electrical response of aortic dECM materials under freeze-dried and rehydrated conditions. **a** Temporal weight change of rehydrated aortic dECM during electrical measurements (12 h). **b** Temporal weight change of freeze-dried aortic dECM during electrical measurements (5 h). **c** Measured open-circuit electrical potential of rehydrated aortic dECM over 10-12 h. **d** Measured open-circuit electrical potential of freeze-dried aortic dECM over 3–5 h. Sample sizes: *n* = 3 per group for electrical measurements

Electrical measurements were reported as hydration-dependent direct-current open-circuit potential (DC-OCP) responses. Weight change profiles during testing (Fig. 5a, b) revealed rapid equilibration of water content in rehydrated samples, with slower stabilization observed in the 4%4D group. DC-OCP measurements (Fig. 5c, d) showed that rehydrated 4%2D samples exhibited higher electrical signal magnitudes than 4%4D samples (*P* < 0.05). Native tissue values were intermediate between groups. Some comparisons were not statistically significant.

## Discussion

### Hydration-dependent modulation of physical properties

A detergent-based protocol was selected for reproducibility and consistency with prior work; while such methods can reduce glycosaminoglycan content and alter ultrastructure, protocol optimization was not the objective of this study. The observed recovery of bulk mass following rehydration reflects hydration-dependent water uptake [54]. However, dimensional changes were not fully reversed. The persistence of increased wall thickness and reduced luminal diameter suggests irreversible microstructural rearrangements, including matrix densification or collapse during freeze-drying [45, 54–57]. These effects are consistent with hydration-mediated osmotic swelling and redistribution of bound versus free water within the matrix. Thus, hydration strongly modulates the physical properties of dECM, yet does not re-establish native geometry. Within this framework, the use of a detergent-based protocol provides a consistent and reproducible processing baseline, enabling isolation of hydration-dependent effects independent of protocol optimization.

### Mechanical anisotropy and water-matrix interactions

Consistent with the anisotropic nature of vascular tissues, circumferential stiffness exceeded longitudinal stiffness at low strain, reflecting elastin-dominated behavior [58–61]. At higher strain, progressive collagen engagement contributed to nonlinear stiffening [59, 62]. These effects are consistent with hydration-induced plasticization, where increased water content enhances molecular mobility, reduces intermolecular interactions, and permits partial fiber reorganization within the collagen–elastin network, thereby modifying viscoelastic coupling.

Although rehydrated dECM exhibited increased stiffness relative to native tissue, measured moduli remained below physiological arterial ranges, indicating that these materials should be interpreted as hydration-modified matrices rather than mechanically equivalent substitutes for native vessels [58, 59]. Differences between 4%2D and 4%4D groups reflect processing-dependent alterations in matrix-water interactions rather than full recovery of native viscoelastic behavior.

Stress-relaxation behavior further demonstrated hydration-dependent modulation. Increased viscous damping in rehydrated samples is consistent with enhanced water content influencing matrix mechanics [60–62].

Vascular tissues experience inherently multiaxial loading with coupled circumferential and longitudinal stresses. Here, uniaxial testing is used as a controlled framework to isolate hydration-dependent material responses. Consequently, the reported properties should be interpreted as reduced-order material descriptors rather than direct predictors of in vivo vascular performance. Accordingly, these results should not be interpreted as predictive of physiological vascular mechanics under multiaxial loading conditions.

### Electrical response and matrix charge distribution

Water content primarily governs matrix plasticization and mechanical compliance, whereas ionic composition modulates charge screening and transport. These effects are coupled but distinct in their contribution to the measured responses.

DC-OCP signals likely arise from ionic gradients, electrokinetic effects, and charge redistribution at the electrode-matrix interface, rather than intrinsic bioelectrical signaling. The observed DC-OCP behavior reflects system-level interactions between water content, ionic mobility, and matrix charge distribution [25, 43, 63–66]. Free water enhances ion transport, while negatively charged GAGs modulate local ionic environments [25, 43, 63, 64, 67]. These processes are governed by coupled electrochemical gradients and matrix-fixed charge density, which together define ion transport behavior within hydrated dECM.

Prolonged detergent exposure in the 4%4D group likely reduced water-binding capacity and matrix continuity, contributing to lower measured electrical signal magnitude [63, 66, 68, 69]. Importantly, these electromagnetic assessments represent hydration-dependent electro-mechanical responses under controlled conditions and should not be interpreted as intrinsic bioelectrical signaling capability [70].

In addition, potential contributions from electrode polarization, interfacial charge accumulation, and evaporation-induced drift during testing cannot be fully excluded. While mass loss was monitored to mitigate hydration changes, these factors may influence absolute signal magnitude and temporal stability. As such, the reported DC-OCP responses should be interpreted as system-level measurements arising from coupled matrix–fluid–electrode interactions rather than as isolated intrinsic material properties.

### Integrated interpretation

In this work, hydration is treated as a state variable in the materials-science sense, i.e., a controllable parameter that serves as a primary determinant of the macroscopic electro-mechanical response under fixed dECM processing conditions. This provides a practical framework for improving reproducibility and comparability across dECM studies through controlled hydration reporting.

In this context, hydration operates alongside established determinants of dECM behavior, including matrix composition, structural organization, and crosslinking density. However, unlike these largely fixed attributes defined during processing, hydration represents a dynamically tunable and environmentally controlled parameter that can be modulated during storage, handling, and testing. This distinction positions hydration as a practical and experimentally accessible variable for standardizing and comparing dECM material responses across studies.

Across physical, mechanical, and electrical measurements, hydration state functioned as a governing and quantifiable state variable that systematically defines the experimentally measured electro-mechanical response of dECM across native, lyophilized, and rehydrated conditions [54, 55]. While rehydration restored bulk water content, dimensional, mechanical, and electrical responses remained distinct from native tissue, underscoring the irreversible influence of processing history.

Collectively, these findings support a materials-centric interpretation of dECM as a hydration-sensitive system rather than as a restored or functionally equivalent tissue replacement [58, 66].

In addition to influencing bulk mechanical and electrical behavior, hydration state likely modulates biological functionality. Water content governs ion mobility, surface charge, and macromolecular spacing within collagen-elastin-GAG networks, affecting cell adhesion, migration, and signaling. Thus, hydration represents not only a governing material's state variable but also a biologically active parameter defining how dECM interfaces with its surrounding biological milieu. Explicit control of hydration conditions is therefore essential when evaluating dECM performance in regenerative or tissue-engineered applications, consistent with hydration-induced plasticization effects.

From an application perspective, physiologically relevant hydration conditions correspond to isotonic ionic environments and full matrix saturation, whereas deviations from these conditions during processing or testing can substantially alter measured responses. The present results highlight that even nominally ‘rehydrated’ states may not replicate native hydration distributions, emphasizing the need for standardized hydration protocols in dECM characterization.

More broadly, these findings suggest that hydration should be treated as a controlled experimental parameter, analogous to strain rate or temperature, and explicitly defined and reported in dECM studies. Adoption of such practices would improve reproducibility, enable more meaningful cross-study comparisons, and support the rational design and evaluation of dECM-based materials for biomedical applications.

## Conclusions

The mechanical and electrical responses of aortic ECM materials are strongly influenced by hydration state and processing history. In native tissue, water-matrix interactions contribute to load damping, viscoelastic behavior, and charge distribution within this biopolymer network. Decellularization and subsequent lyophilization substantially alter these interactions by removing free and bound water and modifying matrix architecture, resulting in pronounced changes in measured physical, mechanical, and electrical responses.

Controlled rehydration induced systematic modulation of dECM behavior, restoring bulk water content and altering stress-strain response, viscoelastic relaxation, and electrical signal magnitude; however, these responses remained distinct from those of native tissues. These findings indicate that processing-induced structural changes impose irreversible constraints on dimensional and mechanical behavior.

Importantly, the observed electrical responses reflect system-level interactions between water content, ionic mobility, and matrix charge distribution, rather than intrinsic bioelectrical function. Analogously, mechanical responses should be interpreted as material-level behavior arising from altered matrix-fluid interactions, instead of native aortic performance. Together, these findings support a materials-centric framework in which the dECM is treated as a hydration-sensitive system governed by environmental state and processing history, consistent with recent materials-focused studies on bio-derived matrices [71–73].

From a design perspective, these findings emphasize the importance of explicitly controlling and reporting hydration conditions during dECM processing and characterization. Effective strategies should consider the balance between free and bound water, matrix porosity, and compositional alterations introduced during decellularization, rather than relying solely on post-processing rehydration to restore native structure.

Beyond materials behavior, this study demonstrates the feasibility of sourcing dECM from abattoir-derived byproducts within a circular bioeconomy framework. Near-complete utilization of collected tissue reduced disposal requirements and environmental impact while providing a consistent materials source for experimental investigation.

In summary, hydration functions as a governing state variable that defines the experimentally measured electro-mechanical behavior of dECM materials. By distinguishing system-level material responses from claims of native tissue equivalence, this work provides a more reproducible and mechanistically grounded framework for evaluating decellularized biomaterials.

## Supplementary information

Supplementary Table 1

## Data Availability

Data will be made available upon reasonable request.
