# Supplementary material for: Hydration as a governing state variable in the mechanical and electrical response of decellularized extracellular matrix materials
Source: J Mater Sci Mater Med. 2026 May 30;37(1):98. doi: 10.1007/s10856-026-07075-6 (PMC13433499; doi:10.1007/s10856-026-07075-6)
Supplement: Supplementary file 1 [file 10856_2026_7075_MOESM1_ESM.docx]

**Supplementary Table 1.** Representative livestock populations and estimated organ/tissue byproduct generated at the Abu Dhabi Automated Slaughterhouse, with associated incineration fees. Values represent operational estimates based on slaughterhouse records and routine disposal practices; actual quantities and fees may vary with animal size, processing conditions, and market rates.

| Livestock Type | Breed | Animals Processed per Day | Organ/Tissue  Byproduct | Total Byproduct Weight (kg) per Animal | Incineration Fee (AED/kg) |
| --- | --- | --- | --- | --- | --- |
| Sheep | Awassi (Najdi) | 15-25 | Eyeballs, aortas, stomachs, intestines, lungs, kidneys, bladders, etc. | 5-15 | 15.2 |
| Goat | Damascus (Shami) | 5-10 | Eyeballs, aortas, stomachs, intestines, lungs, kidneys, bladders, etc. | 5-20 | 15.2 |
| Cattle | Holstein-Friesian | 6-8 | Eyeballs, aortas, stomachs, intestines, lungs, kidneys, bladders, etc. | 15-35 | 25.0 |
| Camel | Majaheem | 4-6 | Eyeballs, aortas, stomachs, intestines, lungs, kidneys, bladders, etc. | 25-55 | 30.0 |
